# Validation of the subjective spine value in patients with adult idiopathic scoliosis

**DOI:** 10.1007/s00402-025-06120-3

**Published:** 2025-11-18

**Authors:** Vincent J. Leopold, Thilo Khakzad, Paul Köhli, Rebecca Hoehl, Robert K. Zahn, Matthias Pumberger, Bernhard U. Hoehl

**Affiliations:** 1 Center for Musculoskeletal Surgery, Charité - Universitätsmedizin Berlin, Charitéplatz 1, 10117 Berlin, Germany; 2 Department of Orthopaedic Surgery, Hospital for Special Surgery, Weill Cornell Medicine, New York City, NY USA; 3 Department of Oral and Maxillofacial Surgery, Charité - Universitätsmedizin Berlin, Augustenburger Platz 1, 13353 Berlin, Germany; 4 Center for Musculoskeletal Surgery, Charité - Universitätsmedizin Berlin, Augustenburger Platz 1, 13353 Berlin, Germany

**Keywords:** Subjective spine value, Adult idiopathic scoliosis, ODI, COMI-back, SRS-22, PROM

## Abstract

**Introduction:**

Patient-reported outcome measures are essential tools in clinical decision-making and research. Multi-item scores are time-consuming to collect and evaluate leading to bias due to missing data. This effect is intensified in vulnerable patient groups with reduced mental health, such as patients with adult idiopathic scoliosis (AdIS). The subjective spine value (SSpV), as a single-item value, assesses spinal function with one question: “What is the overall percent value of your spine if a completely normal spine represents 100%?”. The SSpV was previously validated in a variety of specific spinal disorders. To date, no study assessed the SSpV in patients with AdIS. The hypothesis was that the novel single-item score SSpV would correlate with the established Oswestry disability index (ODI), Core Outcome Measures Index for the back (COMI-back), and Scoliosis Research Society Score (SRS-22) questionnaire in patients with AdIS.

**Materials and methods:**

This cross-sectional study prospectively included 59 patients with AdIS between 04/2022 and 12/2023. The patients completed a questionnaire containing SSpV, ODI, COMI-back, and SRS-22. Spearman's rank correlation coefficient was used to evaluate the correlation between SSpV and the established questionnaires separately. Ceiling and floor effects were evaluated.

**Results:**

The patients were mainly female (female: n = 46, 78%; male: n = 13, 22%) with a median age of 30 years (interquartile range: 23–37 years). The Cobb angle ranged from 11° to 89° (mean: 42.3°; SD: 19.0°). SSpV correlated significantly separately with ODI (*rs* = − 0.487, *p* ≤ 0.001), COMI-back (*rs* = − 0.540, *p* ≤ 0.001), and SRS-22 (*rs* = 0.626, *p* ≤ 0.001). Floor and ceiling effects were low (SSpV: 1%–3%; ODI: 2%–1%; COMI-back: 3%–2%; SRS-22: 1%–3%).

**Conclusions:**

The novel single-item score SSpV validly represents the established multi-item ODI, COMI-back, and SRS-22 in patients with AdIS.

## Introduction

Adult idiopathic scoliosis (AdIS) is characterized by the development of a three-dimensional deformity of the spine involving alterations in shape, size, and position of vertebral bodies, thorax, and trunk [22]. While some evidence exists to guide treatment in adolescent patients with idiopathic scoliosis, recommendations are lacking for these patients when they become adults. In patients with AdIS, the indication for surgery is based additionally on the patient’s complaints [1, 2, 7]. To cover all aspects and complaints of the spine, several patient-reported outcome measurements (PROMs) have been established. In the process of adapting questionnaires to the multi-layered patients’ needs, well-known scores like the Oswestry disability index (ODI) and the Core Outcome Measures Index for the back (COMI-back) were developed. The most widespread scoliosis-specific questionnaire is the Scoliosis Research Society Score (SRS-22) [5]. Originally Haher et al. published it in 1999 containing 24 items [12]. In three major updates, it was condensed to the 22-item version [14]. The current edition contains 5 domains: Function, pain, self-image, mental health (5 questions each), and satisfaction/dissatisfaction with management (2 questions) [4]. The answers were given in multiple-choice method representing a score from 1 (worst) to 5 (best). For evaluation, the mean values were calculated. It is validated across cultural settings [31] and multiple languages [3, 8, 14, 17, 23, 33]. The mean time to complete the questionnaire is indicated as 5.2 ± 3.0 min [25].

Answering multi-item PROMs is time-consuming and may lead to missing data causing bias [21, 26]. This effect is intensified in vulnerable patient groups with reduced mental health such as patients with AdIS [26, 29]. To overcome these limitations and to maximize efficiency and response rates, single-item scores were established in musculoskeletal surgery to evaluate the major joints of extremities e.g. subjective shoulder value (SSV), subjective hip value (SHV) or subjective knee value (SKV) [11, 13, 15, 24, 27, 28]. These single-item scores are defined as the patient’s subjective assessment of their perceived joint function in which a score of 100% represents a full function. Regarding the spine, the subjective spine value (SSpV) was proven valid in spine patients affected by degenerative pathologies, tumor, infection, and trauma. The individual SSpV correlated with the commonly used COMI-back and ODI significantly in all four cohorts [16].

As scoliosis is a special entity where patients suffer a vast variety of complaints containing back pain, body image issues, and self-confidence [32], it is unclear whether the single-item SSpV can sufficiently represent the function of the spine in every dimension. We, therefore, aimed to assess the SSpV and its validity in patients with AdIS. We hypothesized that the SSpV would correlate with the scores of the commonly used COMI-back, ODI, and SRS-22.

## Materials and methods

### Study design

We conducted a prospective, single-center cohort study. Before the initiation of the study, the study protocol was approved by the local Ethics Committee (EA2/078/20). All patients signed informed consent.

We included 59 consecutive adult patients with a primary diagnosis of AdIS who presented to our academic outpatient clinic for spine-specific complaints between 04/2022 and 12/2023.

Included were patients with a primary diagnosis of AdIS, who were over the age of 18 and provided a complete spine-specific questionnaire. Exclusion criteria were prior spinal surgery, age younger than 18, and language barrier resulting in an inability to fill out the questionnaire.

### Study protocol

A diagnosis of AdIS was based on the patient’s history, clinical examination, and radiological findings on standing a.p. radiographs using low-dose biplanar stereo radiography (EOS Imaging, Paris, France). The spinal deformities in AdIS patients were classified according to the Lenke classification (Lenke, Betz et al. 2001) using the Phönix-PACS software (Phönix-PACS GmbH, Freiburg im Breisgau, Germany) by one trained investigator.

### Questionnaire design

All participants completed a spine-specific questionnaire independent of the treating physician before clinical examination. The questionnaire contained basic information (sex, age, body weight, body size), SSpV, ODI (Version 2.0), COMI-back, and SRS-22 [9, 14, 18].

The wording of SSpV was: “What percentage is the function of your spine if the normal function of a healthy spine is 100%? (or: How many euros is your spine worth if a normal spine is worth 100 euros?)” [16].

### Validity

Validity describes whether an instrument is capable of making an abstract concept measurable [30]. As previously shown, the ODI, COMI-back, and SRS-22 are validated and well-established scores to measure spine-specific function [9, 14, 19]. Therefore, we calculated the correlation between the aforementioned multi-item scores and the SSpV for testing validity. Since the scores were not normally distributed variables, Spearman's correlation coefficient (rs) was used. Depending on the correlation coefficient rs, the correlations were interpreted as excellent (0.81 to 1.00); very good (0.61 to 0.80); good (0.41 to 0.60); fair (0.21 to 0.40); and poor (0.00 to 0.20) [10]. Bland–Altman analysis was performed by plotting the differences between the methods against their averages and calculating the limits of agreement [6].

### Data analysis

Descriptive statistics (Frequency rates, means, standard deviation (SD), median, interquartile range (IQR), and floor/ ceiling rates) were utilized to describe baseline patient characteristics. The significance level was set at α < 0.05. Frequency rates, means, and ranges were utilized to describe baseline patient characteristics. The normal distribution was tested using the Shapiro–Wilk test. For correlation, Spearman’s correlation coefficient was used in cases of violation of the normal distribution. Microsoft Excel (Microsoft Corp. Released 2016. Excel Professional for Windows, Version 16.16.2. Redmond, WA: Microsoft Corp.) was used for documentation of the collected data. The collected data were analyzed using IBM SPSS 25 (IBM Corp. Released 2017. IBM SPSS Statistics for Windows, Version 25.0. Armonk, NY: IBM Corp.).

## Results

We included 59 patients with complete datasets (female: n = 46, 78%; male: n = 13, 22%). The median age was 30 years (IQR: 23–37 years) and body mass index ranged from 16.3 kg/m2 to 31.9 kg/m2 (mean: 21.9 kg/m2; SD: 3.3 kg/m2). According to Lenke classification, there were 20 (33.9%) type 1 curves; 1 (1.7%) type 2 curve; 6 (10.2%) type 3 curves; 4 (6.8%) type 4 curves; 18 (30.5%) type 5 curves and 10 (16.9%) type 6 curves. The Cobb angle ranged from 11° to 89° (mean: 42.3°; SD: 19.0°).

SSpV ranged from 5 to 100% (mean: 64%; SD: 20.2%), ODI from 0 to 72% (mean: 21.2%; SD: 15.6%), COMI-back from 0 to 9.7 (mean: 3.9; SD: 2.2) and SRS-22 from 2.0 to 4.3 (mean: 3.3; SD: 0.6). Floor and ceiling effects were low (SSpV: 1%–3%; ODI: 2%—1%; COMI-back: 3%–2%; SRS-22: 1%–3%).

### Oswestry disability index (ODI)

The total score of ODI showed a good correlation with the SSpV (*rs* = − 0.487, *p* ≤ 0.001). The highest correlation was detected in the ODI questions (ODI_Q) personal care (washing, dressing, etc.) (ODI_Q2), lifting (ODI_Q3), sitting (ODI_Q5), and social life (ODI_Q9) (Table 1). Sex life (ODI_Q8) did not reach the level of significance, being an optional question [9].

**Table 1:** Spearman’s rank correlation coefficients (*rs*) between Subjective Spine Value (SSpV) (100% = normal spine) and the Oswestry disability index (ODI) evaluating disability (100% = worst). Statistically significant correlations (p ≤ 0.05) are highlighted in bold.

ODI	Total	ODI_Q1 Pain	ODI_Q2 Personal care	ODI_Q3 Lifting	ODI_Q4 Walking	ODI_Q5 Sitting	ODI_Q6 Standing	ODI_Q7 Sleeping	ODI_Q8 Sex life	ODI_Q9 Social life	ODI_Q10 Traveling
rs	− 0.487**	− 0.348**	− 0.394**	− 0.471**	− 0.295*	− 0.406**	− 0.292*	− 0.286*	**− **0.174	− 0.374**	− 0.301*
Sig. (2-tailed)	≤ 0.001	0.007	0.002	≤ 0.001	0.023	0.001	0.025	0.028	0.216	0.004	0.022
N	59	59	59	59	59	59	59	59	52	58	58

ODI_Q1 = ODI Question 1; * *p* ≤ 0.05; ** *p* ≤ 0.01; N = Number

**Fig. 1:**
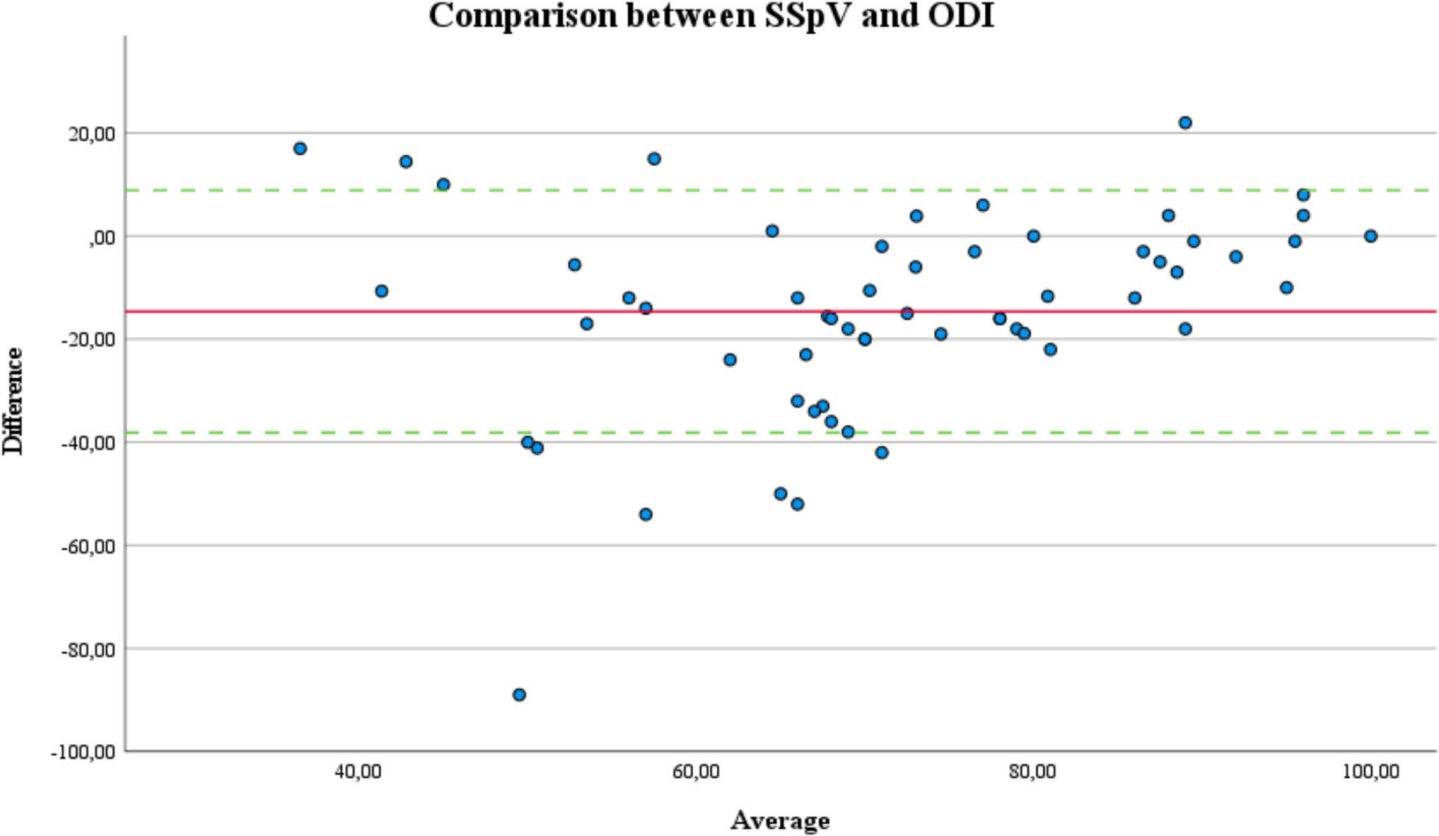
Bland–Altman analysis scatterplot for the precision of agreement is shown for the comparison of the Subjective Spine Value (SSpV) to the Oswestry disability index (ODI) after adjustment of the scale to 0 (worst) to 100 (best). The red line indicates the bias, green lines demonstrate ± 1.96 times the standard deviation around the bias

In Bland-Altmann analysis, the majority of score differences (mean: -14.6) are within the demonstrated 95% limits (-38.1 to 8.9) of agreement. Figure 1 illustrates the precision of agreement between SSpV and ODI after adjusting the original scale from 0 (worst) to 100 (best) for comparability).

### Core outcome measures index for the back (COMI-back)

The total score of COMI-back showed a good correlation with SSpV (*rs* = − 0.540, *p* ≤ 0.001). SSpV did not correlate with the amount of leg pain (COMI_Q1b) in our cohort of patients with AdIS (Table 2). In 11 cases the indicated leg pain (COMI_Q1b: median: 2; interquartile range: 0–4) exceeds the back pain (COMI_Q1a: median: 4.5; interquartile range: 3–6).

**Table 2:** Spearman’s rank correlation coefficients (*rs*) between Subjective Spine Value (SSpV) (100% = normal spine) and the Core Outcome Measures Index for the back (COMI-back) (0 = best; 10 = worst). The higher value from question 1a and question 1b forms the pain value (Question 1). Statistically significant correlations (p ≤ 0.05) are highlighted in bold.

COMI-back	Total	COMI_Q1a Back pain	COMI_Q1b Leg pain	COMI_Q1 Pain	COMI_Q2 Function	COMI_Q3 Symptom-specific well-being	COMI_Q4 General well-being	COMI_Q5 Social disability	COMI_Q6 Work disability
rs	− 0.540**	− 0.392**	**− **0.250	− 0.352**	− 0.530**	− 0.343**	− 0.421**	− 0.515**	− 0.461**
Sig. (2-tailed)	≤ 0.001	0.002	0.063	.006	≤ 0.001	0.008	≤ 0.001	≤ 0.001	≤ 0.001
N	59	58	56	59	58	59	59	57	57

COMI_Q1a = COMI Question 1a; * *p* ≤ 0,05; ** *p* ≤ 0,01; N = Number

**Fig. 2:**
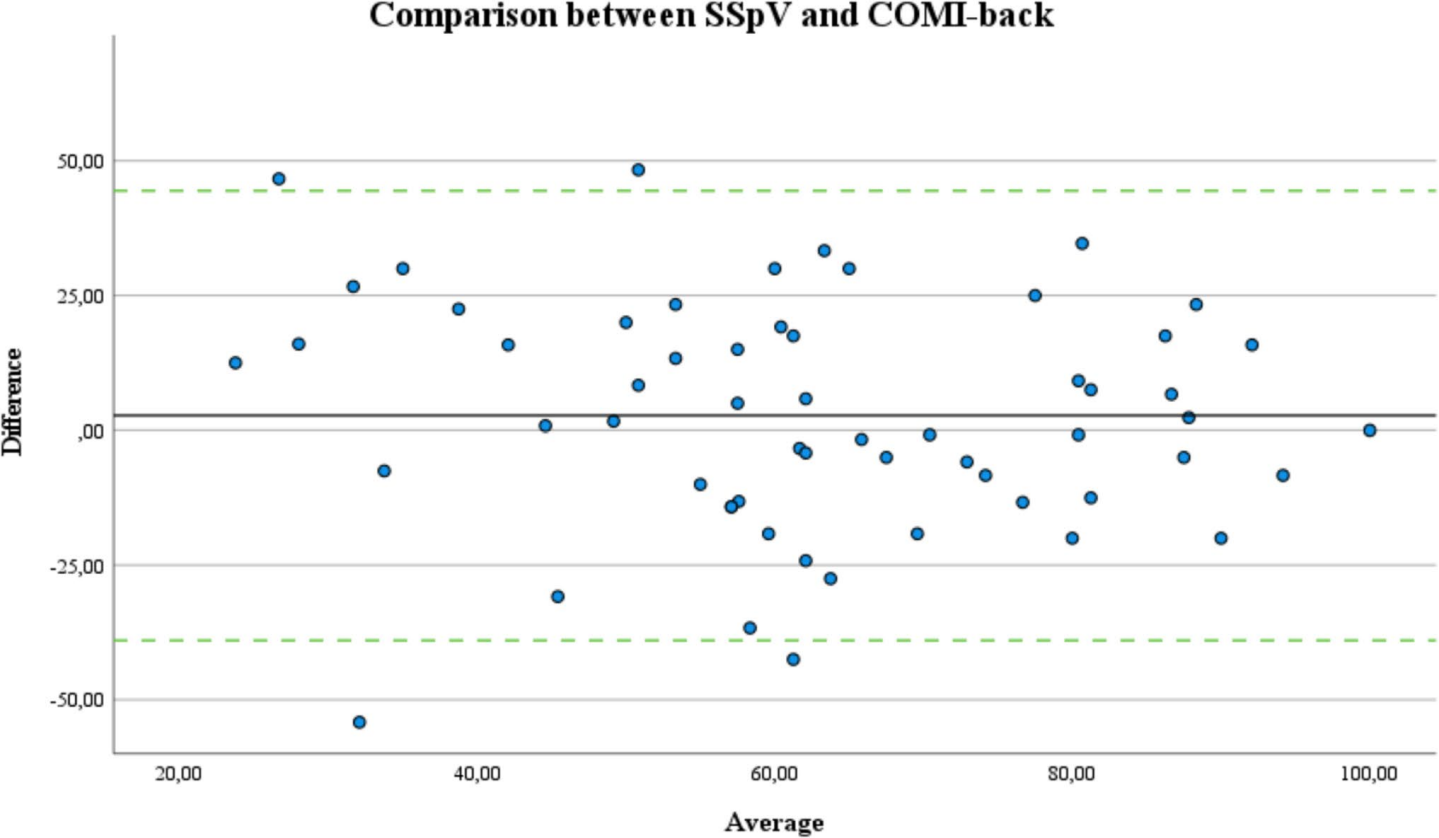
Bland–Altman analysis scatterplot for the precision of agreement is shown for the comparison of the Subjective Spine Value (SSpV) to the Core Outcome Measures Index for the back (COMI-back) after the required adjustment of the scale to 0 (worst) to 100 (best). The red line indicates the bias, green lines demonstrate ± 1.96 times the standard deviation around the bias

The majority of score differences (mean: 2.7) are within the demonstrated 95% limits (-39.0 to 44.4) of agreement, indicating a strong representation of the SSpV for the COMI-back. Figure 2 illustrates the precision of agreement between SSpV and COMI-back after adjusting the original scale from 0 (worst) to 100 (best) for comparability.

### Scoliosis research society score (SRS-22)

The total score of SRS-22 showed a very good correlation with the SSpV (*rs* = 0.626, *p* ≤ 0.001). The domains function (*rs* = 0.519, *p* ≤ 0.001), pain (rs = 0.437, *p* ≤ 0.001), and self-image (rs = 0.567, *p* ≤ 0.001) showed a good correlation with the SSpV. The domains mental health (rs = 0.368, *p* ≤ 0.004) and satisfaction (rs = 0.311, *p* ≤ 0.021) correlated fair. Details are displayed in Table 3.

**Table 3:** Spearman’s rank correlation coefficients (*rs*) between Subjective Spine Value (SSpV) (100% = normal spine) and the Scoliosis Research Society Score (SRS-22) (1 = worst; 5 = best) showed a very good correlation (rs = 0.626,* p* ≤ 0.001). The questions are assigned to the corresponding domains (A)-E)). Statistically significant correlations (p ≤ 0.05) are highlighted in bold.

A) Function	Total A)	SRS_Q5	SRS_Q9	SRS_Q12	SRS_Q15	SRS_Q18
rs	0.519**	0.428**	0.629**	0.335**	0.226	0.139
Sig. (2-tailed)	≤ 0.001	≤ 0.001	≤ 0.001	0.010	0.088	0.308
N	59	58	56	59	58	56

B) Pain	Total B)	SRS_Q1	SRS_Q2	SRS_Q8	SRS_Q11	SRS_Q17
rs	0.437**	0.382**	0.529**	0.120	0.144	0.362**
Sig. (2-tailed)	≤ 0.001	0.003	≤ 0.001	0.369	0.286	0.006
N	59	57	56	58	57	56

C) Self-image	Total C)	SRS_Q4	SRS_Q6	SRS_Q10	SRS_Q14	SRS_Q19
rs	0.567**	0.332*	0.422**	0.641**	0.289*	0.449**
Sig. (2-tailed)	≤ 0.001	0.011	≤ 0.001	≤ 0.001	0.028	≤ 0.001
*N*	59	58	58	56	58	57

D) Mental Health	Total D)	SRS_Q3	SRS_Q7	SRS_Q13	SRS_Q16	SRS_Q20
rs	0.368**	0.242	0.368**	0.386**	0.279*	0.207
Sig. (2-tailed)	0.004	0.067	0.005	0.003	0.034	0.119
N	59	58	57	58	58	58

E) Satisfaction	Total E)	SRS_Q21	SRS_Q22
rs	0.311*	0.256	0.219
Sig. (2-tailed)	0.021	0.059	0.136
N	55	55	48

SRS_Q1 = SRS Question 1; * *p* ≤ 0.05; ** *p* ≤ 0.01; N = Number

**Fig. 3:**
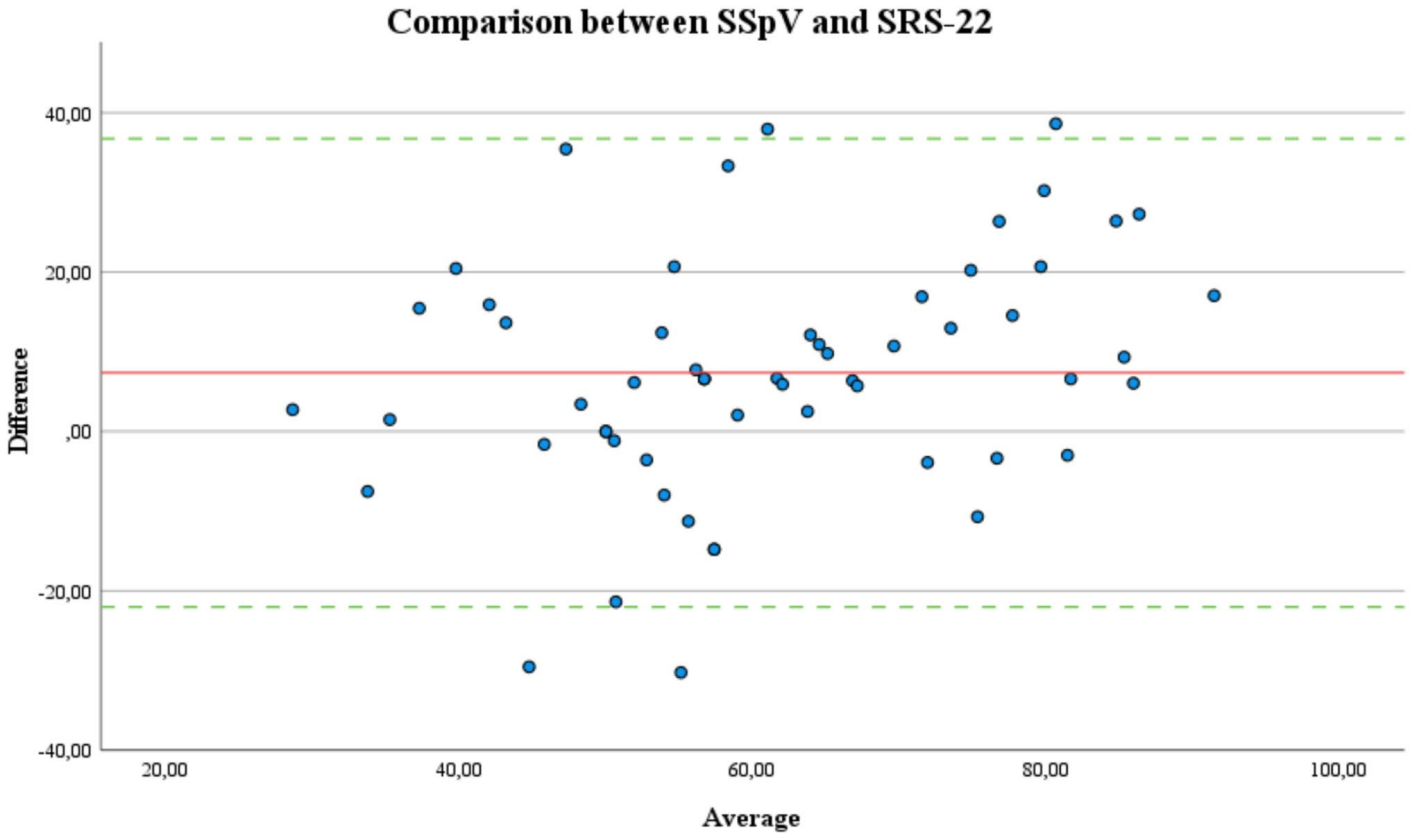
Bland–Altman analysis scatterplot for the precision of agreement is shown for the comparison of the Subjective Spine Value (SSpV) to the Scoliosis Research Society Score (SRS-22) after the corresponding adjustment of the scale to 0 (worst) to 100 (best). The red line indicates the bias, green lines demonstrate ± 1.96 times the standard deviation around the bias

The majority of score differences (mean: − 2.1) are within the demonstrated 95% limits (− 34.7 to 30.5) of agreement, indicating a high validity. Figure 3 illustrates the precision of agreement between SSpV and SRS-22 after adjusting the original scale from 0 (worst) to 100 (best) for comparability.

## Discussion

This was the first study to evaluate a single-item score in AdIS patients. The main finding of the presented study was that the SSpV in the German language showed good to very good correlation at a high level of significance with the established PROMs ODI (“good”, *rs* = − 0.487, *p* ≤ 0.001), COMI-back (“good”, *rs* = − 0.540, *p* ≤ 0.001), and SRS-22 (“very good”, *rs* = 0.626, *p* ≤ 0.001) [10]. Thus, the hypothesis of the study was confirmed.

As they are being assessed independently of a treating physician, PROMs offer a patient-focused perspective on functional status. Since their implementation into clinical practice, they have become essential tools in clinical decision-making as well as clinical research. The relevance of single-item scores and thus the SSpV is particularly emphasized through its ease of administration and interpretation, providing a swift assessment tool in clinical settings where time and resource constraints often limit extensive PROM application. Most established scores, such as the ODI, COMI-back, and SRS-22, are multi-item scales, necessitating considerable time for assessment and evaluation [20], Paranjape, de Araujo et al. 2022). The complexity of these measures can impose constraints on their practical utility in busy clinical settings. In contrast, simple and concise measurement tools enable more efficient data collection and interpretation. This efficiency has fostered the adoption of single-item scores across various disciplines of musculoskeletal surgery, reflecting a shift towards streamlined patient evaluation processes [11, 13, 15, 27].

Plachel et al. validated the SKV in patients with various specific knee pathologies. Their study not only confirmed a high correlation with previously established multi-item scores but also demonstrated superior response rates for the SKV compared to more complex scales [27]. This suggests that the simplicity of single-item scores might significantly enhance patient compliance and data completeness, a crucial factor in busy clinical settings [27].

In this study, the patients presented themselves with a variety of complaints in an academic outpatient clinic specializing in spinal deformities to decide on whether surgery is recommended. Although there is some evidence for guidance in adolescent patients with idiopathic scoliosis, decision-making is more difficult as they transition into adulthood. In patients with AdIS, treatment options remain debatable due to a lack of adequate classifications and recommendations leading to uncertainties in treatment [1, 2, 7]. To improve primary medical care, family doctors and affected patients should be instructed to regularly evaluate the spinal function, considering surgery when dropping below a defined threshold.

In alignment with findings from previous research on single-item scores, studies validating the SKV (SKV vs Knee Injury Osteoarthritis Outcome Score: R = 0.758, *p* < 0.05; SKV vs International Knee Documentation Committee subjective knee form: R = 0.802, *p* < 0.05) and the SHV (SHV vs International Hip Outcome Tool: r = 0.847, *p* < 0.001; SHV vs modified Harris hip score r = 0.832, *p* < 0.001) have reported higher correlations with established scores [13, 15, 27]. This discrepancy may be attributed to the anatomical and functional complexities of the spine compared to the larger joints of the extremities. The spine, unlike large joints such as the knee or hip, comprises a more intricate structural arrangement and is subject to a broader spectrum of biomechanical stresses and pathological conditions. Additionally, in patients with AdIS, the complexity of symptoms extends beyond mere pain to encompass concerns related to body image, as detailed in the SRS-22 score [14, 29]. This inclusion of body image in AdIS patients highlights the multifaceted impact of the condition, which affects not only physical health but also significantly influences psychological well-being, adding another layer of complexity to the assessment. The SRS-22 score provides a comprehensive assessment tool that includes both the physical and psychosocial dimensions of scoliosis, underscoring the unique aspects of AdIS that necessitate consideration in any evaluative measure of spinal function, particularly in a demographic that is often sensitive to changes in appearance and self-perception. Among the PROMs investigated in our study, the correlation between SSpV and the scoliosis-specific SRS-22 was the highest, indicating that the SSpV is capable of capturing this multifaceted impact of AdIS. This observation may be explained by the fact that the simple SSpV consolidates the individual sub-complaints of patients with AdIS according to their subjective weighting in a single score. Among the established assessment tools, the SRS-22 is the only one optimized to capture scoliosis-specific complaints, whereas the ODI and COMI-back were originally developed for the evaluation of general low back pain. Within the subscores of SRS-22, SSpV correlated best with function, pain, and self-image. A comparison of the mental health and satisfaction subscores with other depression scores such as Patient Health Questionnaire-4 (PHQ-4) or Five Well-being Index (WHO-5) could examine the relationship between mental and spine-specific impairment in more detail.

An acknowledged limitation of PROMs includes the potential for floor and ceiling effects, which may restrict their utility by failing to capture further deterioration or improvement beyond the established scoring limits. Remarkably, in the current study, the SSpV exhibited minimal floor and ceiling effects among patients with AdIS, effectively capturing a broad spectrum of spinal function without saturation at the score extremes. This finding aligns with the study by Leopold et al., where the SSpV was validated in a variety of specific spinal pathologies, also demonstrating negligible floor and ceiling effects [16]. These results underscore the robustness of the SSpV in assessing spinal health across varying degrees of severity, enhancing its applicability in clinical and research settings [16].

This study must be interpreted within the context of its limitations. First, the patient cohort was recruited from an academic outpatient clinic specializing in spine-specific complaints and spinal deformity, which may introduce a selection bias. This recruitment strategy limits the generalizability of our findings, as these participants may not accurately represent the broader population of patients with AdIS. Moreover, the participants in this study were relatively young, with a median age of 30 years, which may influence the compensation of symptoms that could manifest differently in older cohorts with similar spinal deformities.

Second, while this study primarily focused on testing the validity of the SSpV, other psychometric properties such as reliability and responsiveness were not assessed. These attributes are critical to understanding the full scope of the score's applicability and effectiveness in clinical practice. Consequently, further research is required to evaluate these properties through longitudinal studies and to examine the responsiveness of the SSpV to therapeutic interventions over time.

Despite these limitations, this study is pioneering in its evaluation of a single-item score to appraise spinal function in patients with AdIS. It offers a foundation for an easily accessible tool to support decision-making on treatment. Subsequent investigations are needed to identify a cut-off value for considering surgery.

## Conclusion

This study successfully demonstrates that the novel single-item score SSpV validly reflects the established multi-item scales on a “good” (ODI and COMI-back) and “very good” (SRS-22) level in patients with AdIS. The SSpV offers a concise and straightforward tool that can be quickly administered and evaluated, potentially facilitating more widespread implementation in routine clinical practice compared to more time-consuming, comprehensive PROMs. This simplicity and efficiency make the SSpV an invaluable tool for decision-making in the shifting landscape of healthcare, where rapid, reliable, and patient-centered outcome measurement is paramount.

## Data Availability

The data that support the findings of this study are available from the corresponding author, B.U.H., upon reasonable request.
